# Changes in Gene Expression Patterns in the Tumor Microenvironment of Head and Neck Squamous Cell Carcinoma Under Chemoradiotherapy Depend on Response

**DOI:** 10.3389/fonc.2022.862694

**Published:** 2022-04-01

**Authors:** Johannes Doescher, Adrian von Witzleben, Konstantinos Boukas, Stephanie E. Weissinger, Gareth J. Thomas, Simon Laban, Jaya Thomas, Thomas K. Hoffmann, Christian H. Ottensmeier

**Affiliations:** ^1^Translational Immunology Group, Institute of Systems, Molecular and Integrative Biology, University of Liverpool, Liverpool, United Kingdom; ^2^Department of Otorhinolaryngology, Head and Neck Surgery, Ulm University Medical Center, Ulm, Germany; ^3^Wessex Investigational Sciences Hub, University of Southampton, Faculty of Medicine, Southampton General Hospital, Southampton, United Kingdom; ^4^Institute of Pathology, Ulm University Medical Center, Ulm, Germany

**Keywords:** head and neck squamous cell carcinoma, chemoradiotherapy, tumor microenvironment, tissue resident memory T cells, gene set enrichment

## Abstract

Chemoradiotherapy (CRT) is a standard treatment for advanced head and neck squamous cell carcinoma (HNSCC). Unfortunately, not all patients respond to this therapy and require further treatment, either salvage surgery or palliative therapy. The addition of immunotherapy to CRT is currently being investigated and early results describe a mixed response. Therefore, it is important to understand the impact of CRT on the tumor microenvironment (TME) to be able to interpret the results of the clinical trials. Paired biopsies from 30 HNSCC patients were collected before and three months after completion of primary CRT and interrogated for the expression of 1392 immune- and cancer-related genes. There was a relevant difference in the number of differentially expressed genes between the total cohort and patients with residual disease. Genes involved in T cell activation showed significantly reduced expression in these tumors after therapy. Furthermore, gene enrichment for several T cell subsets confirmed this observation. The analysis of tissue resident memory T cells (T_RM_) did not show a clear association with impaired response to therapy. CRT seems to lead to a loss of T cells in patients with incomplete response that needs to be reversed. It is not clear whether the addition of anti-PD-1 antibodies alone to CRT can prevent treatment failure, as no upregulation of the targets was measurable in the TME.

## Introduction

Head and neck squamous cell carcinoma (HNSCC) represents the 6^th^ most common cancer worldwide and accounts for approximately 3.8% of all cancer related deaths (1). The most common locations are the oral cavity, the oro- and hypopharynx and the larynx. Carcinoma arising in the nasopharynx are endemic in Asian countries. In general, multimodal treatment comprising surgical resection and adjuvant radiotherapy with or without chemotherapy is indicated except for very early cancers. A significant percentage of patients, however, present in an advanced stage and are too frail to undergo surgery. Additionally, some patients refuse surgery and opt for an organ preserving strategy (2). For this cohort definitive chemoradiotherapy (CRT) with a cumulative cisplatin dose of at least 200 mg/m^2^ is recommended, if toxicity can be tolerated by the patient (3). If treatment fails, salvage surgery is recommended if feasible, as it has been shown to improve significantly overall survival (4). However, some patients are unfit or unwilling to undergo salvage surgery. These patients will mainly be treated with an immunotherapeutic approach with palliative intent according to the latest guidelines (5). Additionally, salvage surgery after radiation treatment harbors a much higher rate of surgical complications and wound healing problems (about 30% of the cases) than upfront surgery (6, 7). These observations show the necessity to improve the efficacy of primary chemoradiotherapy. Therefore, studies combining CRT and immunotherapy, namely immune checkpoint blockade (ICB), have been conducted or are underway with different designs (8, 9). So far, final results have been published only for the JAVELIN Head and Neck 100 trial being the first completed study on a combinatory regimen of CRT and ICB. No benefit for adding anti-PD-L1-antibody avelumab was found for progression-free survival (10). The authors suggest one of the reasons for the unexpected failure of their study might be unfavorable changes in the tumor microenvironment (TME).

The knowledge about direct and late effects of CRT on the TME of HNSCC is very limited and has been studied mainly in experimental settings using animal or *in vitro* models. However, the above-mentioned trials need a better understanding of the effects of CRT to interpret results correctly and improve future trial designs.

Previous work of our group and others identified a T cell subset as possible key candidate for protective immunity in the TME. These cells are characterized by expression of CD8 and CD103 and are now known as tissue resident memory T cells (T_RM_). We were able to show that patients with high density of T_RM_ had better survival outcomes and early data in melanoma hint at T_RM_ being key mediators of the effect of immunotherapy with anti-PD-1 antibodies (11, 12). We therefore hypothesize that CRT is likely to deplete T_RM_ in the TME of patients with impaired response to CRT, either having residual tumor after therapy or relapsing later on. Identifying these patients is crucial and it might be beneficial to expand protective immune cells in these patients before CRT. To test our hypothesis *in vivo* we were able to access a unique sample set of archival tissue biopsies before and after CRT for analysis of changes in the TME.

## Materials and Methods

### Patient Samples

For this retrospective study 30 cases with available paired biopsies before and after definitive CRT were evaluated, resulting in 60 samples. Posttreatment biopsies were taken around 12 weeks after completion of CRT as part of the local standard for response assessment and being in line with the recommendations for the optimal timepoint (13). For initial analysis, patients were divided in two response groups: 1) pathological complete response (CR) if no evidence of tumor after CRT was detected. 2) residual disease (RD) if patients were assessed not tumor-free radiologically and a biopsy after CRT showed remaining tumor cells. Group 1 was subdivided in subsequent analyses into durable complete response (DCR), if no relapse occurred during follow-up, and recurrent/metastatic (RM) if patients were tumor-free after CRT, but encountered a relapse during follow-up. Response assessment was performed by local radiologists and pathologists and discussed in a multidisciplinary team.

Formalin-fixed, paraffin-embedded (FFPE) tumor specimen were collected from the pathology archive at Ulm University Hospital. HPV status was assessed by p16 immunohistochemistry and confirmed by a multiplex HPV-DNA PCR [GP5þ/GP6þ primers followed by Sanger sequencing for HPV typing as described previously (14)]. Cases with both, positive p16 status and presence of HPV high risk type DNA, were considered HPV positive. Serial 5 µm sections were cut and one slide of each sample was stained with hematoxylin and eosin. All samples were re-reviewed by a pathologist (GT) prior to further processing to confirm the abundance or absence of tumor cells and mark regions with high tumor percentage (> 80%). Furthermore, the presence of tumor infiltrating lymphocytes (TIL) was scored according to a previously published scoring system (15). A prominent lymphocytic infiltrate was scored under low-power magnification (× 2.5 objective) as high (diffuse; present in > 80% of tumor/stroma), low (weak/absent; present in < 20% of tumor/stroma), or moderate (patchy; present in 20 – 80% of tumor/stroma). The study was approved by the local ethics committee prior to sample collection (#448/17).

### RNA Expression Analysis

To analyze RNA expression, the HTG EdgeSeq Precision Immuno-Oncology Panel comprising 1392 genes involved in tumor immune interactions, was used. Regions of interest (ROI) were marked on H&E-stained slides and micro dissected from a subsequent fresh, unstained 5 µm cut mounted on a glass slide. ROIs were manually selected for every case with respect to the pathologist’s markings. Tissues were lysed using HTG Lysis Buffer, followed by proteinase K digestion and dilution if necessary; 35 µl of each sample was plated on a 24 well plate and then loaded onto an HTG EdgeSeq platform (HTG Molecular Diagnostics, Inc., Tucson, AZ). Several automated nuclease target protection steps were carried out. First, a quantitative nuclease protection assay (qNPA from HTG) was performed. Nuclease protection probes (NPPs) with universal priming sites (wings) were added to the processed sample lysate solutions. These NPPs were hybridized to their target RNAs producing dsDNA-RNA hybrids. S1 nuclease was added to specifically digest all remaining single stranded nucleic acid chains from excess DNA probes (NPPs) and sample RNAs. S1 nuclease was deactivated, dsDNA-RNA hybrids were melted into single strands, and ssRNA degraded. The resulting DNA probes were quantitatively amplified with polymerase chain reaction adding extended primers (including sequencing primer and sample barcode sequences) for library preparation and cleaned up following a standard clean-up procedure [AMPureXP, PEG8000]. Libraries were then quantified (KAPA Library Quantification Kit for Illumina Platforms; Roche Sequencing and Life Science, Kapa Biosystems, Wilmington, MA), normalized and equimolarly pooled in a 3-pM concentration and loaded on the Illumina NextSeq500 for single-read deep sequencing [Read1: 50 bp, Index1: 6 bp, Index2: 6 bp]. Sequencing base calls were converted into FASTQ and demultiplexed using module bcl2fastq2/2.18 on IRIDIS HPS (UoS) with the option “–barcode-mismatches 0”. FASTQ files were subsequently parsed on HTG Edge parser software and produced a gene expression count matrix.

### Data Analysis and Statistics

Results were normalized using package DESeq2 on R version 4.1.1 and RStudio version 1.4.1717 for Mac (16). Heatmaps were built with the pheatmap package using unsupervised hierarchical clustering *via* Euclidean distance (17). Volcano plots were created with package EnhancedVolcano (18). Principal component analyses were done with PCAtools (19). Gene expression results were corrected for multiple testing using the Benjamini-Hochberg method and differential gene expression was considered significant only with a false discovery rate (FDR) < 0.05. The complete R script can be found on GitHub (20). For immune signature analysis the software platform HTG REVEAL was used. These signatures were developed using the xCell algorithm and have been validated for the HTG EdgeSeq Precision Immuno-Oncology Panel (21, 22). There are 23 signatures for immune or stromal cell types available. Additionally, 3 different types of immune signatures can be calculated; a Stroma Score, an Immune Score and a Tumor Micro Environment (TME) Score. The Stroma Score includes typical stromal cell signatures, i.e. adipocytes, endothelial cells and fibroblasts, whereas the Immune Score is calculated out of immune cell signatures. The TME Score, finally, is a combination of the Stroma and Immune Score. For comparisons of score values before and after therapy a Wilcoxon matched-pairs signed rank test was applied. Results for immune signatures were considered significant with p < 0.05. Figures and statistical comparisons were done with Prism 9 for Mac (GraphPad Software).

## Results

### Patient Characteristics

The cohort comprised 30 patients with advanced stage HNSCC of mainly stage IV. Most patients were treated with a platinum-based combined CRT, two patients received mitomycin C and one patient cetuximab as they were too frail for a platinum-containing regimen. Radiation was delivered in fractions of 2 Gy by IMRT (intensity-modulated radiation therapy) technique. The median applied radiation dose was 68.6 Gy and the majority of patients received therapy as planned with only one patient terminating treatment early due to complications. Patients were biopsied before treatment and a median of 12 weeks after the last dose of radiotherapy as part of the local response evaluation standard in our center in Ulm, Germany. The post-therapy biopsy was taken either from areas suspect for residual cancer tissue in a post-treatment CT-scan or from the former tumor localization in case of complete response. The median follow-up time was 44 months with 77% of patients being either lost to follow-up (n=15) or deceased (n=8) before reaching 5 years. 56.7% were oropharyngeal cases with 47.1% being HPV positive as assessed with p16 status and demonstration of the presence of HPV-DNA. Durable response to CRT was defined as pathological and radiological complete response to the primary treatment with no relapse during the time of follow-up (CR). All patients in this group were under follow-up for at least 2.7 years and most of them for over 4 years. None of the clinical parameters were significantly associated with response to CRT, which reflects the homogeneity of the cohort (**Table 1**).

**Table 1:** Patient characteristics at initial diagnosis and treatment parameters.

Characteristic	DCR (n = 11; 36.7%)	RD + RM (n = 19; 63.3%)	p-Value*
**Gender**			0.850
Male	9 (81.8%)	15 (78.9%)	
Female	2 (18.2%)	4 (21.1%)	
**Age (average in years)**	61.82	63.26	0.631**
**Smoking status**			0.367
Current smoker	8 (72.7%)	10 (52.6%)	
Former smoker	1 (9.1%)	6 (31.6%)	
Never smoker	2 (18.2%)	3 (15.8%)	
**Alcohol consumption**			0.666
Current heavy drinker	2 (18.2%)	3 (15.8%)	
Former heavy drinker	3 (27.3%)	4 (21.1%)	
Moderate daily drinker	3 (27.3%)	3 (15.8%)	
Occasional drinker	3 (27.3%)	6 (31.6%)	
Never drinker	0	3 (15.8%)	
**Tumor site**			0.500 0.637
Oropharynx	7 (63.6%)	10 (52.6%)	
HPV16/p16 positive	4 (57.1%)	4 (40%)	
HPV16/p16 negative	3 (42.9%)	6 (60%)	
Hypopharynx	3 (27.3%)	3 (15.8%)	
Larynx	1 (9.1%)	4 (21.1%)	
Oral cavity	0	2 (10.5%)	
**cT-classification**			0.126
cT1-2	2 (18.2%)	0	
cT3-4	9 (81.8%)	19 (100%)	
**cN-classification**			0.698
cN0	3 (27.3%)	3 (15.8%)	
cN1	1 (9.1%)	3 (15.8%)	
cN2-3	7 (63.6%)	13 (68.4%)	
**Grading**			0.408
G1	0	2 (10.5%)	
G2	7 (63.6%)	13 (68.4%)	
G3	4 (36.4%)	4 (21.1%)	
**Clinical stage**			0.367
II	1 (9.1%)	0	
III	1 (9.1%)	4 (21.1%)	
IVA	8 (72.7%)	11 (57.9%)	
IVB	1 (9.1%)	4 (21.1%)	
**Chemotherapy**			0.482 0.793***
Cisplatin	11 (100%)	16 (84.2%)	
+ Carboplatin	0	1 (6.3%)	
+ 5-FU	0	1 (6.3%)	
Mitomycin C	0	2 (10.5%)	
Cetuximab	0	1 (5.3%)	
Total cisplatin dose (mean ± SD)	229.1 ± 40.4 mg/m^2^	224.4 ± 48.6 mg/m^2^	
**Radiation dose** **(mean ± SD)**	69.3 ± 1.12 Gy	64.9 ± 10.73 Gy	0.093**

*Correlation of clinical parameters and response using chi-square test or Fisher’s exact test depending on expected cell counts.

**Correlation of age and radiation dose and response using unequal variance t-test.

***Correlation of cisplatin dose and response using equal variance t-test.

Percentage is calculated within the response group. DCR, durable complete response; RD, residual disease; RM, recurrent/metastatic disease.

### Histological Assessment of the Immune Infiltrate

TIL status could be assessed on 30/30 pretreatment samples and 6/30 posttreatment samples with residual tumor (**Table S1**). On the pretreatment samples the majority had a low TIL abundance, reflected in a score of 1. A score of 2 and 3 was found in 7 and 5 patients, respectively. Within the HPV positive group, 3 patients were TIL^low^, 1 patient was TIL^moderate^ and 4 patients TIL^high^, which left only one HPV negative case TIL^high^. Analysis of the TIL score in RD after CRT showed remaining low or even lower TIL scores than pretreatment. Taken together, more than half of the patients had very few TILs present before CRT and CRT reduced TIL presence in RD even further (**Figure S1**).

### Differential Gene Expression

**Figure 1:**
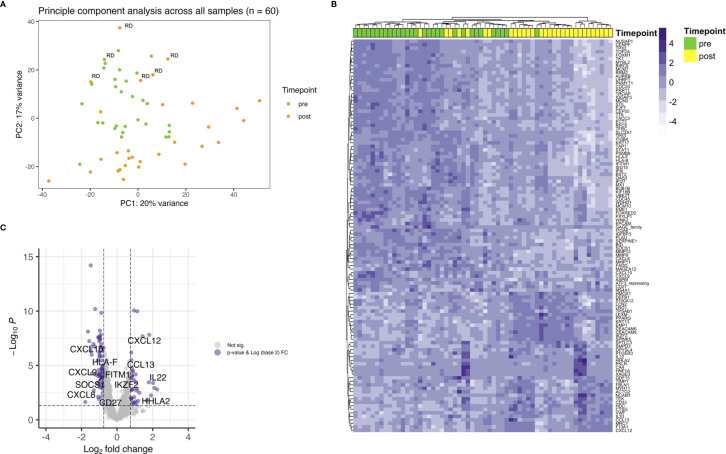
Showing differential gene expression for the whole cohort. **(A)** PCA plot indicating clustering of pretreatment samples (green) and posttreatment samples (orange). Within the green cluster posttreatment samples with RD are present. **(B)** Heatmap depicting unsupervised hierarchical clustering using Euclidian distance for genes with expressed with an FDR < 0.05 and a log2 FC over 0.75. **(C)** Up- and downregulation of T cell related genes after CRT.

First, all samples were analyzed together and differential expression was calculated between before and after treatment biopsies. A principal component analysis (PCA) revealed that pretreatment samples cluster together with posttreatment samples of patients with residual tumor (RD). Most posttreatment samples of patients with pathological complete response after therapy clustered regardless if they developed any recurrence later on (**Figure 1A**). The top genes that drove the PCA were SERPINE1 (fold change (FC) 0.6) and KRT13 (FC 4.3) in PC2. Selection for FDR < 0.05 and a log2 FC of < -0.75 and > 0.75 revealed 112 differentially expressed genes (**Figure 1B**). The top upregulated genes were HNF1A (FC 4.8), KRT13 (FC 4.3), IL22 (FC 4.1), T cell inhibiting HHLA2 (FC 3.7) and CXCL12 (FC 3.5). Members of the cancer testis antigen GAGE family were top downregulated genes after CRT (FC 0.3), followed by SLC2A1 (0.3), IGFBP3 (0.3), MCM2 (FC 0.4) and ISG15 (FC 0.4). Several of the significant differentially expressed genes play a role in mediating T cell functions (**Figure 1C**). Interestingly, among the downregulated genes several are involved in T cell attraction (CXCL9, CXCL10) whereas upregulated HHLA2 inhibits T cell proliferation.

**Figure 2:**
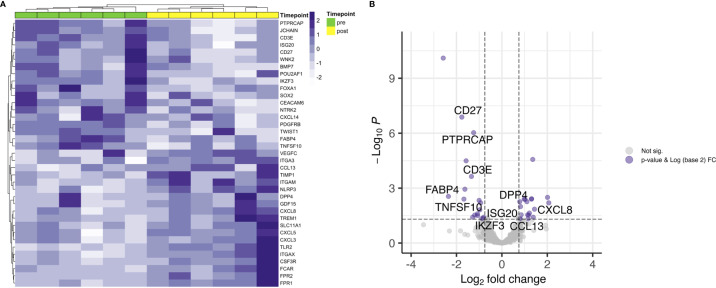
Differential gene expression of RD samples before and after CRT. **(A)** Unsupervised hierarchical clustering using Euclidian distance for genes with expressed with an FDR < 0.05 and a log2 FC over 0.75 shows clear distribution in pre- and posttreatment samples. **(B)** T cell related genes are downregulated after CRT in patients with RD.

Next, differential gene expression between pre- and posttreatment samples limited to patients with residual disease (RD) was analyzed. There were 6 patients with residual vital tumor tissue after completion of CRT. Unsupervised hierarchical clustering for genes with an FDR < 0.05 and a log2 FC < -0.75 and > 0.75 separated samples according to the timepoint of biopsy, i.e. pre- and posttreatment (**Figure 2A**). This is especially interesting when comparing with the whole cohort where no clear clustering towards the timepoint of biopsy could be observed (**Figure 1B**) and pre- and posttreatment samples of the RD subgroup seemed to be similar to all pretreatment samples in PCA (**Figure 1A**). 37 genes were found to be differentially expressed according to the above stated thresholds.

Top upregulated genes were neutrophils recruiting CXCL8 (FC 4.2) and CXCL5 (FC 4.0), pro-inflammatory TREM1 (FC 2.7), matrix metalloproteinase inhibiting TIMP1 (FC 2.6) and GDF15 (FC 2.6). JCHAIN, which is involved in polymerizing IgM and IgA, was found to be the top downregulated gene (FC 0.2). Other top downregulated genes were fatty acid binding protein 4 coding gene FABP4 (FC 0.2), involved in providing fatty acids as an energy resource to T cells, CD27 (FC 0.3), WNK2 (FC 0.3) and neurotrophic tyrosine receptor kinase encoding NTRK2 (FC 0.3). When looking at genes involved in core T cell functions, a decrease in expression of T cell receptor associated CD3E (FC 0.4) and co-stimulatory checkpoint molecule CD27 (FC 0.3) could be observed (**Figure 2B**).

### T_RM_ Gene Signatures

**Figure 3:**
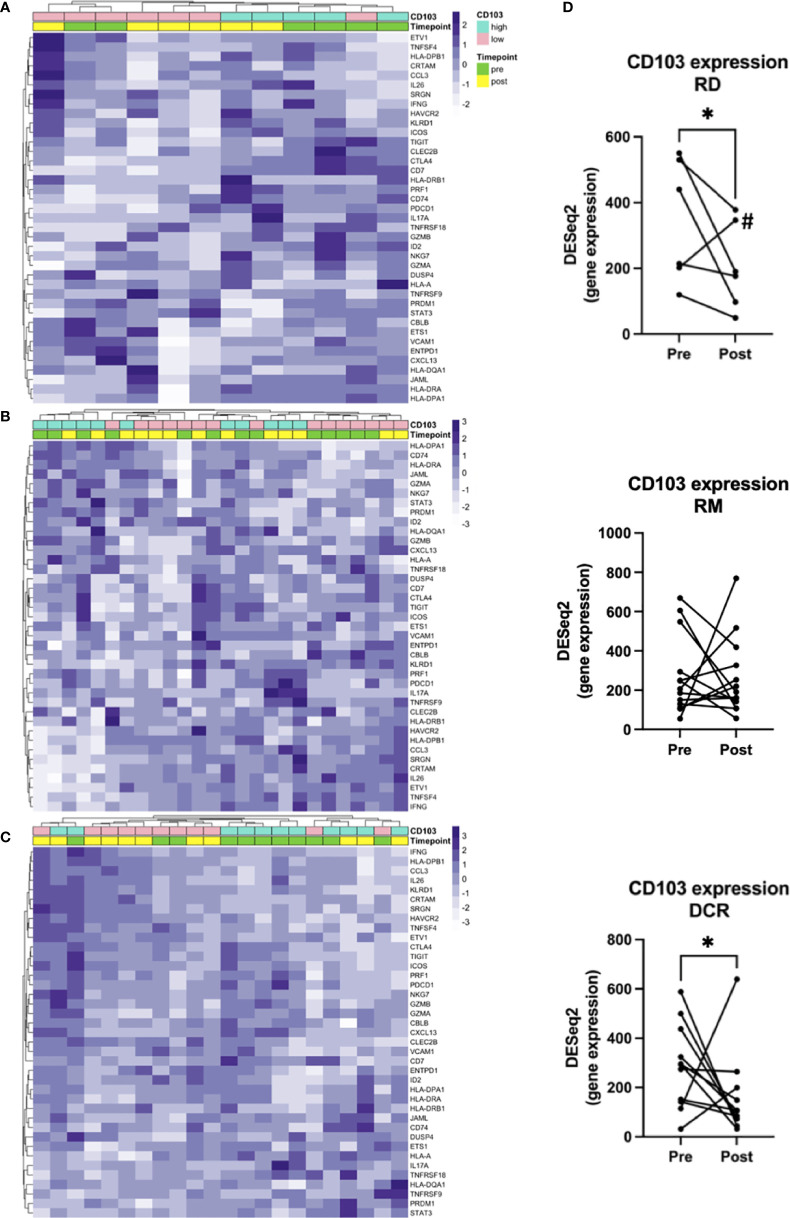
Gene signatures defining T_RM_. Unsupervised hierarchical clustering for **(A)** RD, **(B)** RM and **(C)** DCR indicates no clear changes before and after treatment. **(D)** shows corresponding changes of CD103 expression before and after CRT. # is indicating the Patient treated with RT and cetuximab. * indicates p < 0.05.

To answer our initial question and hypothesis we sought to analyze the gene expression known to be involved in T_RM_. Besides ITGAE, which is the gene for CD103, there have been identified 91 enriched transcripts in previous single cell analyses of lung tumor T_RM_, of which the herein used gene panel covered 39 (**Table S2**) (12). Samples were dichotomized in CD103^high^ and CD103^low^ according to the median log2(CPM+1) value for pre and post treatment samples (**Figure S2**). Applying this gene list, there was no clear clustering of pre- and posttreatment samples, neither for RD and RM (**Figures 3A, B**), nor for patients with durable complete response to CRT (**Figure 3C**). However, unsupervised hierarchical clustering separated cases in CD103^high^ and CD103^low^ especially in the RD subcohort and to a certain extent in the DCR subcohort (**Figures 3A, C**), stating the robustness of the applied gene signature. Additionally, a significant decrease of CD103 expression could be observed for patients with residual tumor (FC 0.6; p = 0.036) and in patients with DCR (FC 2.2; p = 0.009). No clear tendency was seen for patients with known relapse or distant metastasis later in the follow-up (RM) (**Figures 3B, D**). For the target of many immunotherapies, PD-1, no significant changes in expression could be noted for none of the treatment response groups (**Figure S3**). This was also the case for its ligands, PD-L1 and PD-L2 (data not shown).

### Gene Set Enrichment Based Immune Signatures

There is a number of immune phenotype and immune signatures available which have been validated for the Precision Immuno-Oncology Panel. In detail there are signatures available for 23 immune or stromal cell types and 3 different types of immune signatures which were developed using the xCell algorithm (21).

**Figure 4:**
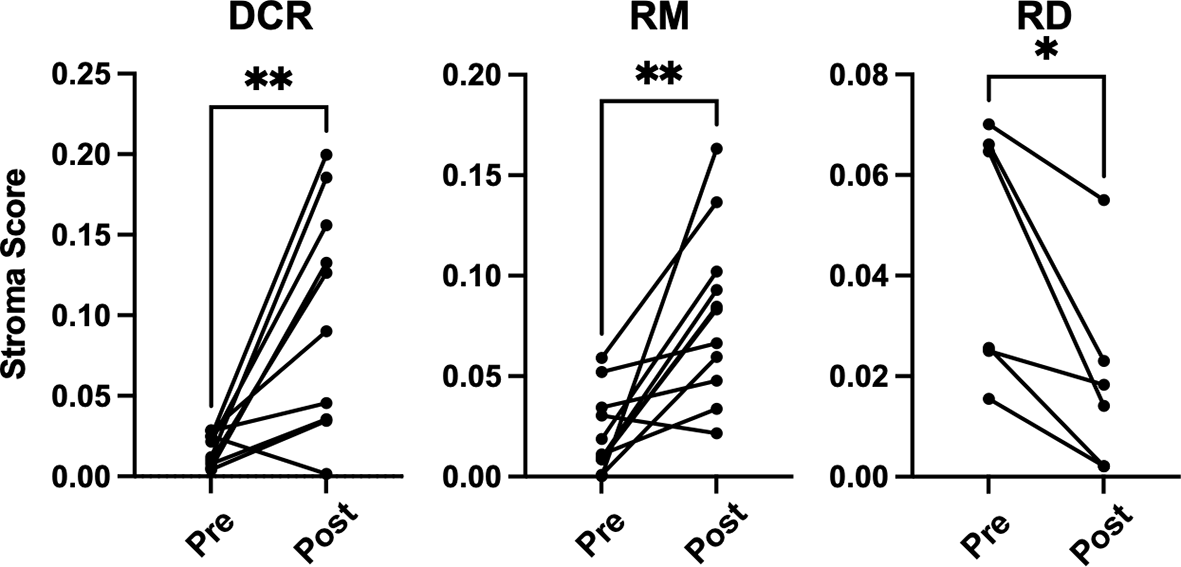
xCell based gene set enrichment analysis shows significant changes of stroma scores with increasing stromal cells in DCR and RM cases compared to decreasing stromal cells in RD cases. * indicates p < 0.05, ** inidcates p < 0.01.

**Figure 5:**
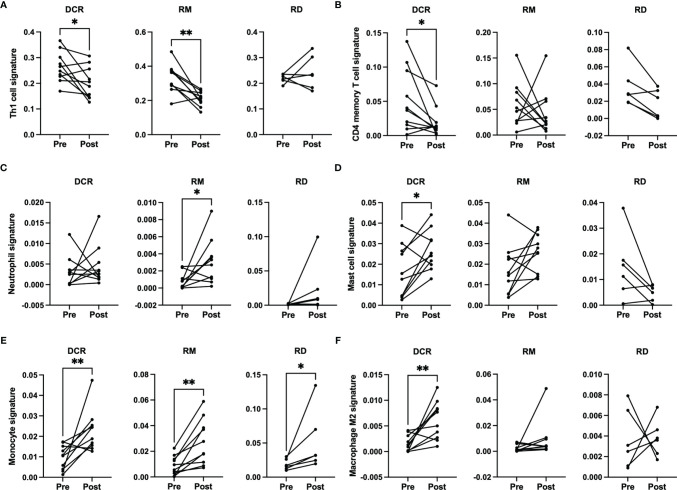
xCell based immune phenotype signature scores for **(A)** Th1 cells, **(B)** CD4 memory T cells, **(C)** neutrophils for RM, **(D)** mast cells, **(E)** monocytes and **(F)** M2 macrophages. * indicates p < 0.05, ** inidcates p < 0.01.

There were no significant changes observed for the Immune and Tumor Micro Environment (TME) Score, but a significant increase of the Stroma Score for patients with pathological complete response (DCR and RM) as opposed to patients with residual tumor cells after CRT, which had a significantly decreased score (**Figure 4**). The Stoma Score takes gene signatures for adipocytes, endothelial cells and fibroblasts into account. When looking at T cell signatures, Th1 cells were decreased after therapy in patients with an initially complete response to CRT (DCR and RM), but not in RD (**Figure 5A**). Interestingly, CD4+ memory T cells were significantly decreased in patients with DCR, but not in patients with RD or a relapse later on (**Figure 5B**).

When looking at cells involved in acute inflammation, a significant increase of neutrophils could be observed for patients with a relapse (RM) (**Figure 5C**). For patients with a durable complete response, an increase of mast cells and monocytes as well as M2 macrophages could be seen (**Figures 5D** **–F**). No significant differences were detectable for the other immune phenotype signatures.

## Discussion

To the best of our knowledge, this is the first study to investigate comprehensive differential gene expression in the TME of head and neck squamous cell carcinoma before and after CRT. In the study a panel consisting of 1392 genes was applied and samples from initial biopsy and around three months after CRT were analyzed and compared. Immune cells are highly sensitive to radiation on the one hand, but adaptive immune activation appears to happen in patients treated with radiation treatment. Our starting question was whether we could identify an effect of CRT on key T_RM_ cells we and others have reported to be protective and to confer a better survival (11, 12). Our central question was therefore whether T_RM_ were involved in the response to CRT and or reduced in patients with residual tumor after treatment (RD) or development of recurrent and/or metastatic disease later on (RM).

First, we examined the overall differentially expressed genes in a pairwise comparison of samples before and after CRT. The expression profile of most pretreatment samples was similar to the expression profile of RD posttreatment as revealed in a principal component analysis. This is not surprising as tumor cells were abundant in the samples at both timepoints, whereas samples of DCR and RM cases had no evidence of tumor cells after treatment. The key driver genes of the components were SERPINE1 (downregulated) and KRT13 (upregulated). SERPINE1 has been proposed as a prognostic marker for progression-free survival of HNSCC patients treated with CRT or radiotherapy. High SERPINE1 expression was significantly associated with the development of metastasis and reduced overall survival. In cell lines it could be shown that high SERPINE1 expression is associated with cisplatin resistance (23). Additionally, there are several reports on incorporation of the gene in predictive and prognostic signatures, where high expression was always associated with either impaired survival or low response to therapy (24–26). Together with our observation that SERPINE1 is downregulated in patients with complete response to CRT, but not in RD patients, this creates an interesting picture and might add to the understanding of resistance to CRT. It should be noted, however, that due to the small sample size and the focus on paired analysis before and after CRT, it was not useful to examine prognostic survival parameters related to individual gene expression in our work. This limitation is also reflected in the lack of significant associations between response and clinical parameters. KRT13 is known to be downregulated in patients with active oral squamous cell carcinoma as compared to dysplasia or normal mucosa (27). So, it is very well possible that after successful tumor clearance and remodeling the overall expression increases again, as observed in this study, and is a marker of response to CRT.

Besides KRT13, HNF1A and IL22 were top downregulated in the overall cohort. HNF1A is located on chromosome 12q24.3 and encodes the transcription factor Hepatocyte nuclear factor 1-alpha (28). The role of the transcription factor in HNSCC is yet to be defined, but for other entities, such as colorectal and esophageal cancer, a correlation with impaired survival and resistance to anticancer drug therapy and radiotherapy could be linked to high HNF1A expression (29, 30). This seems to stand in contrast to our findings as HNF1A was upregulated under CRT when analyzing the overall cohort, but not when focusing on the RD subgroup.

After CRT, tissue regeneration is important and therefore it is of no surprise to find IL22 upregulated after CRT in all patients, but not in patients with residual disease. IL22 is mainly produced by T cells and its receptors are broadly expressed on epithelial cells where a protective role against infections is executed (31). A more worrying finding comes from an *in vitro* study, where IL22 production was elevated in a cancer associated fibroblast culture and treatment of lung cancer cells with the supernatant of this culture resulted in enhanced proliferation and migration (32). However, as in the present study bulk RNA has been analyzed it is not possible to connect IL22 expression to a distinct cell type and attempts with common deconvolution algorithms, such as CIBERSORTx, failed due to the limited number of genes in the panel.

It is well established that T cells are crucial for anti-cancer immunity and abundance of especially CD8+ T cells has been linked to improved survival of HNSCC patients (33). Therefore, it was one of the aims of this study to investigate the expression change of genes which are involved in T cell function under CRT. There were detectable differences in a few genes when looking at the overall cohort and focusing on RD cases. In the posttreatment TME of the overall cohort a decrease as well as an increase in the expression of several chemokines involved in T cell attraction could be observed. In the subgroup analysis of RD, however, there were no mainly T cell attracting chemokines upregulated after CRT, but CXCL8, which is encoding IL-8. IL-8 is known to be involved in mediating inflammation through activation of neutrophils and is upregulated in several cancers including HNSCC (34). Interestingly, this was significantly downregulated in the overall cohort, which consisted mostly of therapy responders. Moreover, a significant downregulation of CD3E and CD27 was present in patients with RD after CRT, but not in the overall cohort. CD3E is expressed specifically on T cells and high expression could be linked to a favorable prognosis (35). In an earlier study on a slightly different cohort, we were already able to show a loss of expression of CD27, which is expressed on T cells and is a co-stimulatory molecule, on protein level (36). This supports the observation made here of a downregulation of these two genes in poor responder under therapy. The loss of T cell signatures in patients poorly responding to CRT could be further demonstrated using the Immunephenotyping Signatures, which were developed and tested for the 1392 gene panel and are based on the xCell gene set enrichment algorithm (21, 22). According to these signatures, CD4 memory T cells were diminished after CRT in responder. Furthermore, lower Th1 cell signatures were counted in patients with complete response to CRT, independent of a later recurrence. Th1 type cells are known to promote anti-tumor immunity upon antigen encounter and infiltration with these cells is associated with good prognosis (37). This might explain the observation herein as the baseline levels of Th1 TIL in responder (DCR and RM) were higher than in RD and Th1 activity was reduced after removal of cancer cells by CRT. Finally, the remodeling of the tissue could be verified by the stroma score, which consists of signatures for adipocytes, fibroblasts and endothelial cells and was significantly increased in DCR and RM patients but not in RD patients.

Earlier work of our group could identify T_RM_ as key candidates for long-lasting protective immunity and it could be shown that they are highly functional when expressing PD-1 and TIM-3 in lung cancer and melanoma (11, 12). Just recently, the question about the role of T_RM_ in HNSCC has been addressed by Ida et al. Although, their definition of T_RM_ as CD8^high^ CD69^high^ was different, they found abundance of these cells to be associated with improved overall survival in a TCGA cohort and an upregulation of several immune checkpoint molecules on CD8^high^ CD69^high^ T cells including PD-1 and TIM-3 in both tissue and peripheral blood. Interestingly, there was no correlation with disease-free survival and infiltration of T_RM_ detectable (38). In the present work no clear regulation of genes related to T_RM_ could be seen for any of the response groups. However, there was a slight loss of CD103 expression in RD patients after CRT, whereas this was not measurable in RM. CD103 expression was also decreased in DCR, which might be due to the fact of complete antigen removal from the tissue. This could indicate that in some patients T_RM_ are depleted by CRT, despite persistence of antigen, which needs to be further studied to protect these patients from a harmful treatment. It has to be noted, that a major limitation of this study is the use of bulk RNA and a focused gene panel. This makes it difficult to deconvolute cell types properly and only the gene enrichment assays specifically tested for the panel produce reliable data. So single cell analysis of the TME before and after therapy might add knowledge to the role of T_RM_ in the response to CRT. But this is not without methodical difficulties, as the tissue sample has to be large enough to isolate a sufficient number of immune cells. In turn, the tight anatomy of the head and neck makes it challenging to justify a larger biopsy after treatment without compromising function. Another approach, potentially more feasible, is the application of imaging mass cytometry. With this technique it is possible to assess up to 40 markers on a small amount of tissue and with spatial resolution. We plan to track our gene expression data using this method in the future. Lastly, it might also be of interest to analyze biopsies of recurrent tumors. Although, this has been done and compared to pretreatment biopsies by other groups, these investigations lacked the step of analyzing the immediate changes in the TME (39). In a planned prospective study building upon our herein presented results, we aim to collect samples longitudinally and include analysis of recurrences to assess changes in the TME over time.

In conclusion, we could find differences in the change of the TME between patients with pathological complete response and impaired response to CRT on a transcriptomic level. The data indicate the formation of an inflamed, but T cell poor TME in patients who poorly respond to CRT and the addition of immune-stimulatory drugs to CRT seems to be justified for patients, who will not respond to CRT alone. But predictive markers to define this group of patients are still lacking and need to be found. Further, the study was not able to find any relevant differences between patients with a durable tumor control and those with a relapse later on. Therefore, this might not be an issue of the TME alone, but more of the global immunocompetence of cancer patients.

## Data Availability Statement

The datasets presented in this study can be found in online repositories. The names of the repository/repositories and accession number(s) can be found below: https://www.ncbi.nlm.nih.gov/geo/, GSE193445.

## Ethics Statement

The studies involving human participants were reviewed and approved by Institutional Ethics Committee of University of Ulm. Written informed consent for participation was not required for this study in accordance with the national legislation and the institutional requirements.

## Author Contributions

Conceptualization, JD and CO. Methodology, KB. Software, JD, AW, and JT. Formal analysis, JD. Investigation, KB, SW, and GT. Resources, CO, SL, and TH. Data curation, JD. Writing—original draft preparation, JD. Writing—review and editing, all authors. Visualization, JD. Supervision, CO and SL. Funding acquisition, CO and JD. All authors have read and agreed to the published version of the manuscript.

## Funding

This research was funded by Deutsche Forschungsgemeinschaft (DFG), project number 451445144.

## Conflict of Interest

JD received reimbursements for advisory boards of MSD and Merck Serono.

The remaining authors declare that the research was conducted in the absence of any commercial or financial relationships that could be construed as a potential conflict of interest.

## Publisher’s Note

All claims expressed in this article are solely those of the authors and do not necessarily represent those of their affiliated organizations, or those of the publisher, the editors and the reviewers. Any product that may be evaluated in this article, or claim that may be made by its manufacturer, is not guaranteed or endorsed by the publisher.
