# Supplementary material for: Changes in Gene Expression Patterns in the Tumor Microenvironment of Head and Neck Squamous Cell Carcinoma Under Chemoradiotherapy Depend on Response
Source: Front Oncol. 2022 Apr 1;12:862694. doi: 10.3389/fonc.2022.862694 (PMC9012140; doi:10.3389/fonc.2022.862694)
Supplement: Supplementary file 1 [file DataSheet_1.pdf]

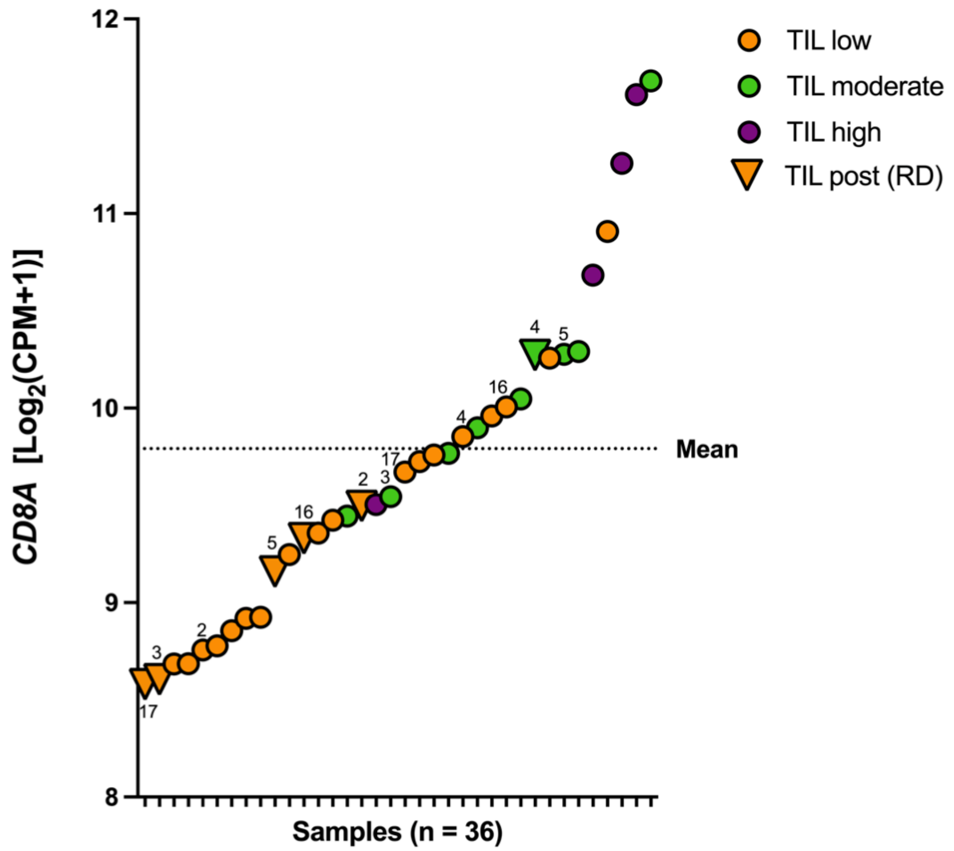

Figure S1. Correlation between CD8A expression and morphological TIL scoring. TIL assessment was only possible for samples with tumour present, which is why for most cases, except for RD, only pretreatment samples are shown. Pairs are indicated with sample IDs.

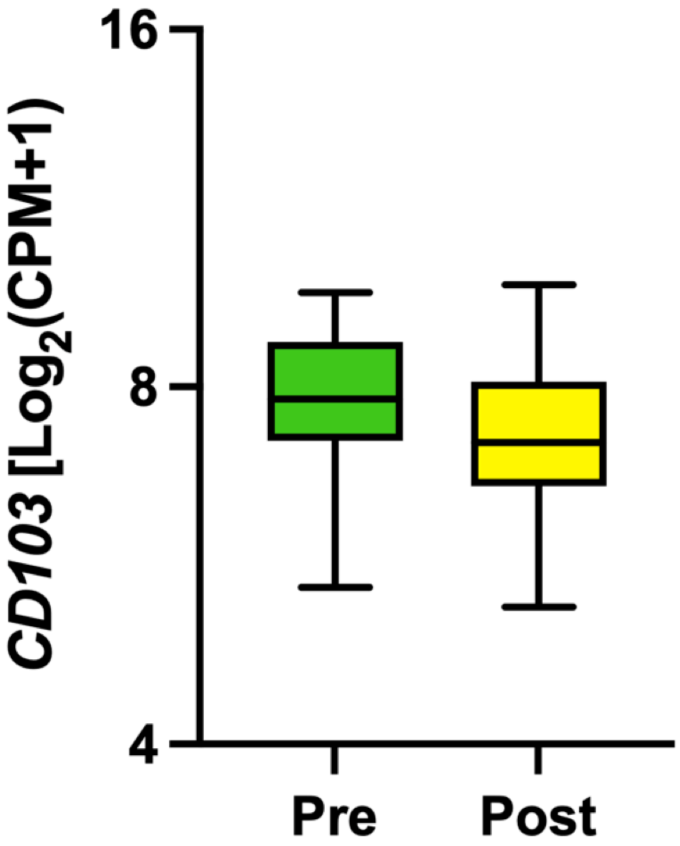

Figure S2. Median CD103 expression before and after CRT across all samples. No significant difference in expression was found using a paired t-test ($p = 0.05$).

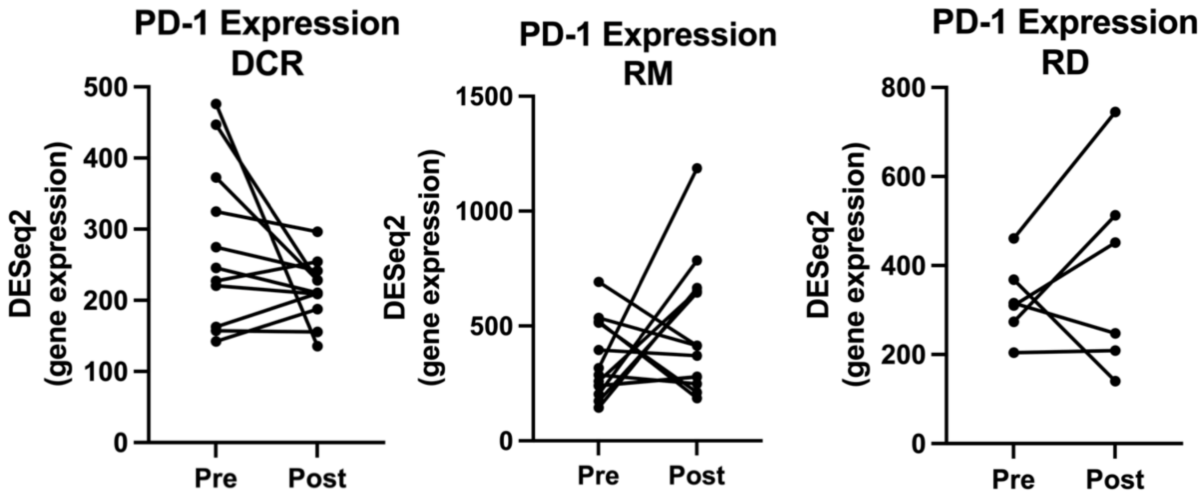

Figure S3. Comparison of PD-1 expression in the TME between the three different response groups. There was no significant change under therapy in any of the groups (p = 0.2 for DCR, p = 0.3 for RM, p = 0.4 for RD).
